# Supplementary material for: Identification of Genomic Characteristics and Selective Signals in a Du’an Goat Flock
Source: Animals (Basel). 2020 Jun 6;10(6):994. doi: 10.3390/ani10060994 (PMC7341327; doi:10.3390/ani10060994)
Supplement: Supplementary file 1 [file animals-10-00994-s001.pdf]

Table S1 Summary of sequencing and mapping results in 15 Du'an goat

Sample ID	SRR ID	Number of clean reads	Number of mapped clean reads	Alignment rate	Duplicate rate	Mean depth (x)
DASY12	SRR11744862	224,011,046	222,318,175	99.24%	45.54%	11.2886
DASY36	SRR11744859	152,650,411	152,225,623	99.72%	44.60%	7.7465
DAG22	SRR11744863	192,433,877	191,901,472	99.72%	44.14%	9.7649
DAG21	SRR11744864	180,898,224	180,183,544	99.60%	45.45%	9.1452
DAG26	SRR11744855	173,916,733	173,481,470	99.75%	43.79%	8.8296
DAG23	SRR11744857	170,095,909	169,596,247	99.71%	43.93%	8.6026
DASY6	SRR11744858	166,240,088	165,743,596	99.70%	45.48%	8.4159
DAG28	SRR11744853	192,612,367	192,005,681	99.69%	45.35%	9.738
DASY18	SRR11744861	181,589,452	181,030,681	99.69%	44.76%	9.2007
DAG32	SRR11744850	217,569,014	216,836,665	99.66%	46.23%	10.6515
DAG27	SRR11744854	191,832,142	191,415,249	99.78%	44.05%	9.7445
DAG29	SRR11744852	227,247,411	226,766,558	99.79%	45.98%	11.4713
DASY20	SRR11744860	168,613,142	168,204,147	99.76%	44.76%	8.5482
DAG31	SRR11744851	185,316,037	184,591,041	99.61%	45.67%	9.3274
DAG25	SRR11744856	198,182,877	197,709,645	99.76%	43.68%	10.0265

Table S2 Inbreeding coefficient (F) for each individual

Sample ID	O(HOM)	E(HOM)	N_SITES	F
DAG21	11454714	11262951	16000064	0.04048
DAG22	11557435	11326650	16097532	0.04837
DAG23	11413773	11309434	16070814	0.02191
DAG25	10995608	11334062	16110117	-0.07086
DAG26	11588615	11316657	16082069	0.05707
DAG27	11385078	11332256	16106331	0.01106
DAG28	11434171	11236731	15962568	0.04178
DAG29	11515042	11215881	15932461	0.06343
DAG31	12179757	11241240	15971463	0.19841
DAG32	11800305	11195655	15903508	0.12843
DASY12	11312371	11327505	16099718	-0.00317
DASY18	11303120	11303913	16063770	-0.00017
DASY20	11561176	11287801	16039265	0.05753
DASY36	12573915	11253239	15988489	0.2789
DASY6	11773326	11213831	15925730	0.11874

Table S3 Runs of homozygosity for each individual

Sample ID	ROH coverage	F_{ROH}
DAG21	1171.04	0.000474837
DAG22	3170.84	0.001285723
DAG23	3494.37	0.00141691
DAG25	2146.44	0.000870346
DAG26	1010.65	0.000409802
DAG27	2223.63	0.000901645
DAG28	1074.65	0.000435753
DAG29	2045.65	0.000829477
DAG31	13468.4	0.005461215
DAG32	<1000	<0.000405484
DASY12	2457.3	0.000996395
DASY18	2102.25	0.000852428
DASY20	10019.9	0.004062905
DASY36	44312.5	0.017967989
DASY6	4561.27	0.00184952

Table S4 Historic effective population size over 1000 generations ago

GenAgo	Ne	dist	r2	r2SD	items
13	55	3748864	0.108892	0.132078	1804806456
15	62	3272928	0.10962	0.132877	1649470761
17	71	2844065	0.110234	0.133469	1498364732
20	81	2459647	0.111178	0.134443	1354418283
23	94	2116476	0.111741	0.135056	1218944811
27	109	1811276	0.112321	0.135651	1088272697
32	127	1540998	0.112974	0.136381	969420091
38	150	1303465	0.113499	0.136865	857908177
45	176	1095284	0.114646	0.13805	751892205
54	210	914235	0.115376	0.138653	656431587
65	251	757794	0.116235	0.139543	569855101
80	303	623660	0.11697	0.140429	490894897
98	368	509561	0.117741	0.141358	417167917
120	446	413425	0.11933	0.142987	351419479
150	544	333182	0.121145	0.145041	292743702
187	668	267070	0.122976	0.147026	241263703
234	817	213284	0.125459	0.149901	196068278
293	994	170185	0.12879	0.153728	156491497
366	1198	136250	0.132837	0.158088	122666053
454	1427	110074	0.137311	0.162981	93910889
553	1674	90372	0.141786	0.167786	69917922
658	1921	75979	0.146205	0.172409	50372552
759	2152	65841	0.14997	0.176278	34740147
847	2346	59013	0.15297	0.179257	22669358
914	2489	54683	0.155163	0.181402	13792284
958	2579	52147	0.156753	0.182938	7584530
983	2633	50822	0.157433	0.18348	3600901
995	2657	50237	0.157724	0.183797	1336185
999	2668	50041	0.157712	0.183761	312169

Table S5 Putatively selected regions in Du'an goat

Chromosome	Start	End	Nucleotide diverstiy	CLR statistic	iHS statistic	Candidate genes
1	44260001	44350000	0.000318	149.7978	NS	LOC102177192, CMSS1
1	46860001	46990000	0.000179	374.4433	NS	
1	50320001	50450000	0.000211	223.0397	NS	CBLB
1	58120001	58250000	7.79E-05	317.6937	NS	USF3, ATP6V1A, NAA50
1	69880001	69950000	0.000314	NS	0.694444	RPL35A, LMLN, IQCG
2	29020001	29230000	0.000282	NS	0.467532	RNF25, LOC102177672, STK36, LOC102169634, ZNF142, PLCD4, TTLL4
2	44160001	44370000	0.000262	258.0465	NS	RAPH1, ABI2
2	44480001	44570000	0.000415	170.6136	NS	NBEAL1
2	75440001	75530000	NS	383.1533	0.527778	
2	77140001	77250000	NS	199.6235	0.546341	
3	23540001	23830000	0.000242	539.9734	0.551724	LOC106501971
3	24840001	24950000	0.0003	NS	0.708333	
3	27940001	28050000	NS	206.1218	0.531792	DMRTB1
3	30180001	30290000	0.000379	178.8822	NS	

3	39120001	39250000	NS	254.0436	0.659091	ROR1
3	56820001	56910000	0.000367	NS	0.454148	
3	58540001	58630000	0.00029	177.866	NS	ADGRL2
3	95760001	95910000	0.000186	318.9708	NS	
4	5140001	5330000	0.000254	213.5754	NS	GALNT11, KMT2C
4	13100001	13270000	NS	255.2734	0.61194	LOC102188562, LOC102188468, LOC102188286, LOC102188017, LOC102187740, LOC102187456, LOC102187191
4	14140001	14250000	0.000321	183.9534	NS	LOC102183208, LOC108635908, LOC108635909, LOC102181470
4	19520001	19730000	0.000239	177.0643	0.75	CHRM2
4	61360001	61530000	0.00022	NS	0.765517	
4	61880001	61970000	0.000228	NS	0.721854	
4	85740001	85910000	NS	155.0298	0.54321	
4	115960001	116090000	NS	218.2651	0.520891	
5	15640001	15730000	0.00011	NS	0.626263	
5	15840001	15930000	0.000226	196.7955	NS	
5	16880001	17170000	NS	793.0345	0.851852	
5	18600001	18830000	7.03E-05	522.6512	0.562963	
5	18940001	19090000	0.000138	357.0315	0.689655	DUSP6, POC1B
5	20700001	20890000	7.65E-05	4.22E+02	NS	KERA, EPYC, LUM, DCN
5	47240001	47490000	2.60E-05	579.2216	NS	HMGA2
5	49660001	49910000	NS	7.43E+02	0.443299	AVPR1A
5	58060001	58350000	NS	3.31E+02	0.600583	LOC102181274, LOC102181570, LOC102181844, LOC102182107, LOC102180572, LOC102182384, LOC102180830, LOC102181102, LOC102181370, LOC102181659, LOC102181931
5	58780001	58930000	0.000406	NS	0.943396	SNRPF, CCDC38, NTN4
5	64500001	64690000	0.000141	NS	0.589041	PMCH, CCDC53, NUP37, PARPBP
5	72720001	72830000	0.000239	177.8766	NS	RBFOX2
6	32480001	32610000	NS	190.9344	0.757962	GRID2
6	35660001	35810000	NS	196.1115	0.708075	
6	36860001	37010000	5.65E-05	NS	0.62069	HERC6, PPM1K, ABCG2
6	54360001	54650000	0.000186	217.3848	0.923664	
6	70640001	70830000	7.29E-05	2.80E+02	NS	KIT
7	10220001	10350000	NS	189.6684	0.543131	
7	22940001	23110000	0.000171	2.98E+02	0.525773	
7	23140001	23310000	NS	287.9299	0.526012	
7	85460001	85550000	0.0004	152.2038	NS	SLC27A6
7	94020001	94110000	0.000334	NS	0.833333	LOC102176031
8	220001	310000	0.000399	NS	0.474359	MFSD14B
8	4220001	4310000	NS	156.7253	0.587719	GALNTL6
8	60740001	60870000	0.000355	NS	0.476563	PAX5
8	70960001	71070000	0.00023	NS	0.928571	

9	9280001	9450000	NS	321.0787	0.867133	IBTK
9	17700001	17850000	NS	250.318	0.648352	
9	18020001	18130000	NS	188.083	0.582857	
9	28860001	29130000	0.000216	485.1031	NS	FOXO3, LACE1
9	56720001	56950000	0.000156	NS	0.89243	ARG1, MED23, ENPP3
9	61260001	61450000	0.000144	234.4024	NS	MTFR2, BCLAF1, PDE7B
9	62180001	62290000	0.000328	NS	0.570707	IL22RA2, IFNGR1
9	66280001	66470000	0.000269	NS	0.92228	
9	76240001	76350000	0.000212	NS	0.592593	ESR1
9	88000001	88110000	0.000269	NS	0.487603	LOC108636750, LOC108636749, PDE10A
10	71480001	71630000	0.000246	NS	0.716981	
10	76020001	76170000	NS	314.7871	0.755639	LOC102178263, LOC108636866, LOC102183422, LOC102182595, LOC102182312, LOC102181504, LOC108636909, LOC102181784
10	79760001	79870000	0.000417	NS	0.477157	PPP1R3E, PABPN1, SLC22A17, RNF212B, LOC102172159, HOMEZ
10	82300001	82570000	0.000123	169.3666	NS	GRAMD2, SENP8, MYO9A
11	69680001	69810000	0.000387	276.5338	NS	ALK
11	78160001	78350000	0.000267	261.0076	NS	SDC1, PUM2
11	78580001	78750000	0.000216	192.1578	NS	TTC32, WDR35
11	95040001	95170000	0.00028	NS	0.518072	NR5A1, NR6A1
12	32040001	32170000	0.000257	NS	0.568966	
12	83880001	84090000	0.000244	191.7419	NS	DIAPH3
13	2760001	2890000	0.000134	214.5475	NS	
13	12140001	12210000	0.000376	1.56E+02	NS	ITIH2, ATP5C1, KIN
13	51480001	51710000	0.000176	320.8437	0.626667	TMEM239, C13H20orf141, VPS16, PCED1A, PTPRA
13	61740001	61990000	6.03E-05	310.0402	0.559211	LOC106502777, BPIFA2
14	15940001	16190000	0.000367	200.4207	0.461774	RPL30, RIDA, ERICH5, MATN2, POP1, NIPAL2
14	35520001	35650000	0.000325	241.6625	NS	EXT1
14	40140001	40310000	0.00027	337.2264	NS	ZC2HC1A, PKIA
14	66940001	67090000	0.000129	186.3097	NS	KIAA0196, NSMCE2
14	85200001	85470000	5.58E-05	1.15E+03	NS	
15	9820001	9910000	NS	172.9861	0.601266	
15	11720001	11850000	NS	161.997	0.597656	
15	13980001	14110000	NS	371.7936	0.463068	
15	32200001	32450000	3.26E-05	NS	0.588235	LOC102177547, LOC102177261, RRM1, STIM1
15	34780001	35050000	0.000406	689.3078	0.776256	LOC102181861, LOC102180484, LOC102180752, LOC102176880, LOC102182689, LOC102181024, LOC102181293, LOC102169612, LOC102169034, LOC102184641, LOC102179935, LOC102182401
15	39800001	40070000	0.000295	NS	0.684492	LOC102177819, ADM, SBF2
15	74360001	74590000	0.000418	280.9516	0.77533	ARHGAP42, PGR
15	81740001	81950000	6.12E-05	158.0948	0.52795	LOC102187333
16	29620001	29770000	NS	171.6605	0.47412	SMYD3

16	32240001	32310000	0.000352	NS	0.495495	SDCCAG8
16	40780001	41090000	7.43E-05	549.9406	NS	ANGPTL7, SRM, UBIAD1, MASP2, EXOSC10, MTOR
16	43160001	43550000	0.000126	172.6358	NS	RERE
16	53600001	53750000	0.000105	213.197	NS	KLHL20, CENPL, ANKRD45, DARS2
16	78880001	79210000	6.81E-05	1020.478	NS	ARL8A, ELF3, LOC102168445, GPR37L1, IPO9, LMOD1, TIMM17A, RNPEP, LOC108637802, PTPN7, NAV1, LOC108637801
17	25460001	25590000	0.000346	531.947	NS	
17	41020001	41250000	0.000262	2.29E+02	0.654545	ABHD18, MFSD8, LARP1B
17	56680001	56950000	NS	951.231	0.964377	FREM3
17	66780001	66910000	0.000121	309.3733	NS	TLR2, RNF175, KIAA0922
18	41260001	41370000	0.000273	2.91E+02	NS	HYDIN
18	41400001	41550000	NS	292.1832	0.456647	PLEKHF1, C18H19orf12, POP4
19	3440001	3670000	NS	455.8072	0.944206	KIF2B
20	20360001	20470000	0.000317	188.262	0.656	PDE4D
20	50280001	50390000	NS	234.8138	0.538462	
21	36480001	36650000	NS	461.9061	0.555556	LOC102171823
21	52720001	52870000	NS	425.632	0.538206	
22	16180001	16370000	9.21E-05	283.4265	NS	ZNF852, ZNF502, ZNF501, KIAA1143, LOC102177570, KIF15
22	28560001	28750000	0.000333	NS	0.861386	PPP4R2, GXYLT2
22	51680001	51870000	0.000125	NS	0.707143	LOC102181444, MAP28, LOC102170003, LOC102171106, CDC25A, MAP4
23	10200001	10310000	0.000408	200.1074	0.517857	RBM24, STMND1
23	38260001	38330000	0.000407	NS	0.464646	STK38, KCTD20
23	39960001	40070000	0.000294	NS	0.608696	C23H6orf106
23	48300001	48570000	NS	163.7392	0.95749	KHDRBS2
24	62040001	62330000	NS	1087.407	0.969697	LOC102184299, LOC108633803, LOC108633802, SERPINB7, LOC102181552, LOC102181826, SERPINB11
25	1120001	1190000	0.000377	NS	0.490741	IFT140, TMEM204, CRAMP1
25	34420001	34530000	0.000383	NS	0.5625	LOC102190953, LOC102181832
25	41520001	41650000	0.000235	335.3452	NS	GRIFIN, IQCE, TTYH3, LFNG
26	15060001	15150000	NS	178.2399	0.481013	PNLIPRP3
26	30420001	30590000	0.000387	NS	0.624434	PKD2L1, BLOC1S2, CWF19L1, ERLIN1, CHUK
26	33340001	33530000	0.000357	NS	0.869863	SLIT1, LCOR
26	42280001	42390000	NS	179.3297	0.51773	MINPP1
28	23820001	24350000	NS	389.5261	0.956044	
28	32140001	32270000	0.000169	272.445	NS	LOC102172123
28	36120001	36270000	NS	166.3166	0.548023	LGALS8, EDARADD, HEATR1

Table S6 Nonsynonymous SNP under selection in Du'an goat

Chromosome	Position	Frequency	Gene.refGene	ExonicFunc.refGene	AAChange.refGene
2	29195151	1	ZNF142	nonsynonymous	XM_013972691.2:c.A2291G:p.H764R
3	28020806	1	DMRTB1	nonsynonymous	XM_018046498.1:c.A248T:p.Q83L
3	28020820	1	DMRTB1	nonsynonymous	XM_018046498.1:c.A262G:p.T88A
4	5179086	1	KMT2C	nonsynonymous	XM_018046667.1:c.A1655G:p.E552G
4	5234959	1	KMT2C	nonsynonymous	XM_018046679.1:c.G5392A:p.G1798S
4	5236364	1	KMT2C	nonsynonymous	XM_018046679.1:c.A6797G:p.H2266R
4	5252005	1	KMT2C	nonsynonymous	XM_018046679.1:c.T9956C:p.V3319A
4	5253175	1	KMT2C	nonsynonymous	XM_018046679.1:c.G11126C:p.G3709A
4	5310637	1	GALNT11	nonsynonymous	XM_013963616.2:c.A226G:p.N76D
4	13117233	0.833333	LOC102188562	nonsynonymous	XM_013963481.2:c.G407A:p.S136N
4	13146377	1	LOC102188468	nonsynonymous	XM_005679632.2:c.A59C:p.K20T
4	13163955	0.966667	LOC102188286	nonsynonymous	XM_013963480.2:c.T911G:p.L304W
4	13164069	0.961538	LOC102188286	nonsynonymous	XM_013963480.2:c.G797A:p.R266Q
4	13164106	0.964286	LOC102188286	nonsynonymous	XM_013963480.2:c.G760T:p.A254S
4	13207093	0.933333	LOC102187740	nonsynonymous	XM_013963478.2:c.T574A:p.W192R
4	13207519	0.966667	LOC102187740	nonsynonymous	XM_013963478.2:c.C148T:p.L50F
4	13207561	1	LOC102187740	nonsynonymous	XM_013963478.2:c.G106T:p.V36F
4	13207605	1	LOC102187740	nonsynonymous	XM_013963478.2:c.G62A:p.G21D
4	13207659	1	LOC102187740	nonsynonymous	XM_013963478.2:c.G8C:p.G3A
4	14182184	1	LOC102183208	nonsynonymous	XM_005679620.2:c.A637G:p.I213V
4	14182652	0.833333	LOC102183208	nonsynonymous	XM_005679620.2:c.A169G:p.M57V
4	14201422	0.857143	LOC108635908	nonsynonymous	XM_018047511.1:c.A362G:p.H121R
4	14235741	0.833333	LOC108635909	nonsynonymous	XM_018047512.1:c.C710T:p.S237F
4	14235777	0.833333	LOC108635909	nonsynonymous	XM_018047512.1:c.A674T:p.K225M
5	58117005	1	LOC102181844	nonsynonymous	XM_005680454.2:c.A364G:p.M122V
5	58274941	0.866667	LOC102181370	nonsynonymous	XM_005680624.2:c.C446T:p.T149I
5	58299945	0.866667	LOC102181659	nonsynonymous	XM_013963976.1:c.T523C:p.W175R
6	36961369	0.933333	PPM1K	nonsynonymous	XM_013964547.2:c.T157C:p.C53R
6	70777648	1	KIT	nonsynonymous	NM_001285724.1:c.G1284T:p.Q428H
7	85500751	0.966667	SLC27A6	nonsynonymous	XM_018050707.1:c.G656A:p.R219Q
7	94105802	1	LOC102176031	nonsynonymous	XM_005682333.3:c.A719G:p.N240S
9	9389415	1	IBTK	nonsynonymous	XM_018052914.1:c.A392G:p.D131G
9	9389699	1	IBTK	nonsynonymous	XM_018052914.1:c.C108G:p.S36R
9	61391034	1	BCLAF1	nonsynonymous	XM_005684799.3:c.T1402G:p.L468V
9	62183820	0.821429	IL22RA2	nonsynonymous	XM_005684844.2:c.G98A:p.R33Q
9	62203779	0.966667	IFNGR1	nonsynonymous	XM_005684807.3:c.A733G:p.I245V
10	76044106	0.857143	LOC108636866	nonsynonymous	XM_018053775.1:c.G130A:p.V44M
10	76060923	0.821429	LOC102183422	nonsynonymous	XM_005685424.3:c.C620G:p.T207S
10	76084639	0.9	LOC108636909	nonsynonymous	XM_018053808.1:c.C253A:p.P85T
10	76156605	0.833333	LOC102181504	nonsynonymous	XM_005685419.2:c.G919A:p.G307R
10	82510647	1	MYO9A	nonsynonymous	XM_018053559.1:c.A5020G:p.I1674V
11	78628624	1	TTC32	nonsynonymous	XM_005686979.3:c.G87C:p.Q29H

11	78628843	1	TTC32	nonsynonymous	XM_005686979.3:c.C306G:p.N102K
11	78636071	1	TTC32	nonsynonymous	XM_005686979.3:c.T779C:p.V260A
12	83936722	1	DIAPH3	nonsynonymous	XM_018056821.1:c.A3215G:p.Q1072R
13	12168557	0.966667	ITIH2	nonsynonymous	XM_005687846.3:c.G2489A:p.R830H
13	12186526	1	KIN	nonsynonymous	XM_018056964.1:c.A703G:p.T235A
13	51686580	1	PCED1A	nonsynonymous	XM_005688214.3:c.G5A:p.G2E
13	51691194	1	PCED1A	nonsynonymous	XM_005688214.3:c.A1345G:p.M449V
14	15963101	0.933333	MATN2	nonsynonymous	XM_018058266.1:c.G943A:p.A315T
14	15985489	1	MATN2	nonsynonymous	XM_018058271.1:c.G942C:p.K314N
14	16114231	0.933333	POP1	nonsynonymous	XM_018058275.1:c.C409T:p.H137Y
15	34873557	1	LOC102181861	nonsynonymous	XM_005701482.2:c.G151T:p.V51F
15	34874074	1	LOC102181861	nonsynonymous	XM_005701482.2:c.A668G:p.H223R
15	34874345	0.966667	LOC102181861	nonsynonymous	XM_005701482.2:c.A939T:p.Q313H
15	34886980	1	LOC102180484	nonsynonymous	XM_005701478.2:c.C190T:p.L64F
15	34911572	0.966667	LOC102176880	nonsynonymous	XM_005702116.2:c.T163C:p.C55R
15	34912314	1	LOC102176880	nonsynonymous	XM_005702116.2:c.A905G:p.Q302R
15	34930862	0.964286	LOC102182401	nonsynonymous	XM_005701484.2:c.A794G:p.H265R
15	34930930	1	LOC102182401	nonsynonymous	XM_005701484.2:c.A862G:p.M288V
15	34930933	1	LOC102182401	nonsynonymous	XM_005701484.2:c.A865C:p.I289L
15	34947136	0.866667	LOC102182689	nonsynonymous	XM_005701485.2:c.T62C:p.V21A
15	34956371	0.866667	LOC102181024	nonsynonymous	XM_005701480.2:c.C59T:p.P20L
15	34956683	0.866667	LOC102181024	nonsynonymous	XM_005701480.2:c.G371A:p.R124H
15	35022070	1	LOC102169034	nonsynonymous	XM_005689798.2:c.A374G:p.Q125R
15	35022166	1	LOC102169034	nonsynonymous	XM_005689798.2:c.G470A:p.R157Q
16	40996359	1	MTOR	nonsynonymous	NM_001285748.1:c.G5507C:p.G1836A
16	41035026	1	EXOSC10	nonsynonymous	XM_005690686.3:c.G1654A:p.V552I
16	53744101	1	DARS2	nonsynonymous	XM_005690891.3:c.G1214A:p.S405N
16	78882534	1	LOC102168445	nonsynonymous	XM_018059973.1:c.A4085G:p.Q1362R
16	78906071	0.966667	PTPN7	nonsynonymous	XM_018060865.1:c.A680G:p.K227R
16	78920814	0.966667	GPR37L1	nonsynonymous	XM_018060867.1:c.A1358G:p.H453R
16	78921351	0.966667	GPR37L1	nonsynonymous	XM_018060867.1:c.G821A:p.R274Q
16	78923923	0.966667	GPR37L1	nonsynonymous	XM_018060867.1:c.G188T:p.G63V
16	78954168	1	NAV1	nonsynonymous	XM_018060868.1:c.G54C:p.Q18H
16	79022775	1	NAV1	nonsynonymous	XM_018060869.1:c.A3094G:p.I1032V
16	79176284	1	LOC108637802	nonsynonymous	XM_018060878.1:c.A619G:p.T207A
16	79176320	1	LOC108637802	nonsynonymous	XM_018060878.1:c.A583G:p.M195V
17	56737811	1	FREM3	nonsynonymous	XM_005691247.2:c.T5129G:p.F1710C
17	56737826	1	FREM3	nonsynonymous	XM_005691247.2:c.G5114A:p.G1705E
17	56737877	1	FREM3	nonsynonymous	XM_005691247.2:c.C5063G:p.T1688R
17	56737975	1	FREM3	nonsynonymous	XM_005691247.2:c.C4965G:p.S1655R
17	56738103	1	FREM3	nonsynonymous	XM_005691247.2:c.A4837G:p.T1613A
17	56738142	1	FREM3	nonsynonymous	XM_005691247.2:c.A4798G:p.K1600E
17	56738226	1	FREM3	nonsynonymous	XM_005691247.2:c.G4714A:p.A1572T
17	56738483	1	FREM3	nonsynonymous	XM_005691247.2:c.A4457G:p.Q1486R

17	56738543	1	FREM3	nonsynonymous	XM_005691247.2:c.G4397A:p.R1466H
17	56738565	1	FREM3	nonsynonymous	XM_005691247.2:c.T4375C:p.F1459L
17	56738588	1	FREM3	nonsynonymous	XM_005691247.2:c.T4352G:p.I1451S
17	56738690	1	FREM3	nonsynonymous	XM_005691247.2:c.C4250T:p.S1417L
17	56738693	1	FREM3	nonsynonymous	XM_005691247.2:c.C4247G:p.T1416S
17	56738783	1	FREM3	nonsynonymous	XM_005691247.2:c.G4157C:p.R1386T
17	56738807	1	FREM3	nonsynonymous	XM_005691247.2:c.G4133A:p.R1378Q
17	56738996	1	FREM3	nonsynonymous	XM_005691247.2:c.T3944C:p.M1315T
17	56739802	1	FREM3	nonsynonymous	XM_005691247.2:c.G3138T:p.E1046D
17	56739890	1	FREM3	nonsynonymous	XM_005691247.2:c.C3050A:p.P1017H
17	56739926	1	FREM3	nonsynonymous	XM_005691247.2:c.A3014C:p.Q1005P
17	56740577	1	FREM3	nonsynonymous	XM_005691247.2:c.A2363G:p.Q788R
17	56741556	1	FREM3	nonsynonymous	XM_005691247.2:c.T1384C:p.C462R
17	56741981	1	FREM3	nonsynonymous	XM_005691247.2:c.A959C:p.Q320P
17	56742326	1	FREM3	nonsynonymous	XM_005691247.2:c.C614T:p.T205M
17	56742821	1	FREM3	nonsynonymous	XM_005691247.2:c.C119G:p.P40R
17	66803578	0.933333	KIAA0922	nonsynonymous	XM_018061620.1:c.A3623G:p.K1208R
17	66865412	1	TLR2	nonsynonymous	NM_001285603.1:c.T47C:p.V16A
17	66866104	1	TLR2	nonsynonymous	NM_001285603.1:c.A739G:p.I247V
17	66878671	1	RNF175	nonsynonymous	XM_013970492.2:c.C816A:p.D272E
18	41335783	1	HYDIN	nonsynonymous	XM_018063160.1:c.C11711T:p.A3904V
22	16233954	1	LOC102177570	nonsynonymous	XM_005695602.3:c.G25A:p.E9K
22	16253655	1	LOC102177570	nonsynonymous	XM_005695603.3:c.T535G:p.F179V
22	16262453	0.933333	LOC102177570	nonsynonymous	XM_005695603.3:c.A2138T:p.E713V
22	51689364	0.933333	MAP28	nonsynonymous	NM_001285546.1:c.T468G:p.H156Q
22	51709595	0.933333	LOC102171106	nonsynonymous	XM_005696010.3:c.G127A:p.D43N
23	48441325	0.933333	KHDRBS2	nonsynonymous	XM_005696178.3:c.T734C:p.L245P
24	62051844	1	LOC102184299	nonsynonymous	XM_018039670.1:c.A649G:p.N217D
24	62054214	1	LOC102184299	nonsynonymous	XM_018039670.1:c.C242T:p.A81V
24	62168180	1	SERPINB7	nonsynonymous	XM_005697316.2:c.A82G:p.M28V
24	62271122	1	LOC102181552	nonsynonymous	XM_005697318.2:c.A483C:p.E161D
24	62285846	0.892857	LOC102181826	nonsynonymous	XM_005697319.2:c.A5G:p.D2G
24	62290261	0.866667	LOC102181826	nonsynonymous	XM_005697319.2:c.G214A:p.G72S
25	1185386	1	CRAMP1	nonsynonymous	XM_018040033.1:c.G1552A:p.G518R
25	1187247	0.866667	CRAMP1	nonsynonymous	XM_018040033.1:c.G2317A:p.A773T
25	41636278	1	GRIFIN	nonsynonymous	XM_018040010.1:c.G113A:p.S38N
26	30501459	0.866667	CWF19L1	nonsynonymous	XM_005698326.2:c.A178G:p.I60V
26	33355411	0.866667	LCOR	nonsynonymous	XM_005698279.2:c.G3029A:p.G1010E
26	33355927	0.866667	LCOR	nonsynonymous	XM_005698279.2:c.G2513C:p.S838T
28	32200466	1	LOC102172123	nonsynonymous	XM_005699073.2:c.A67G:p.T23A
28	32201472	0.9	LOC102172123	nonsynonymous	XM_005699073.2:c.T455C:p.V152A
28	32201535	0.9	LOC102172123	nonsynonymous	XM_005699073.2:c.C518G:p.A173G
28	32201559	0.9	LOC102172123	nonsynonymous	XM_005699073.2:c.C542G:p.T181R
28	32201862	0.9	LOC102172123	nonsynonymous	XM_005699073.2:c.C845A:p.T282K

28	36159042	1	HEATR1	nonsynonymous	XM_018042235.1:c.T1949G:p.I650S

Table S7 Exonic indels under selection in Du'an goat

**Chromosome**	**Position**	**Frequency**	**Gene.refGene**	**ExonicFunc.refGene**	**AAChange.refGene**
2	44262704	1	RAPH1	frameshift_insertion	XM_018060606.1:c.2337dupC:p.R779fs
4	14182547	1	LOC102183208	nonframeshift_insertion	XM_005679620.2:c.273_274insTTC:p.T92delinsFT
4	14201567	0.857143	LOC108635908	nonframeshift_insertion	XM_018047511.1:c.216_217insACA:p.S73delinsTS
4	14220818	1	LOC102181470	frameshift_insertion	XM_013963476.2:c.165dupC:p.L55fs
4	14236203	1	LOC108635909	frameshift_insertion	XM_018047512.1:c.247_248insC:p.L83fs
4	14236282	0.9	LOC108635909	frameshift_insertion	XM_018047512.1:c.168dupC:p.M57fs
11	78628559	1	TTC32	frameshift_deletion	XM_005686979.3:c.23_24del:p.L8fs
28	32201351	0.9	LOC102172123	frameshift_insertion	XM_005699073.2:c.335dupT:p.V112fs

Supplementary Note: Key parameters for software
BWA-MEM: -t 12 -M -R '@RG\\tID:%s\\tLB:%s\\tPL:ILLUMINA\\tSM:%s'.

Picard: SortSam I=$BAM/%s.bam O=$BAM/%s.sort.bam SORT_ORDER=coordinate and MarkDuplicates I=$BAM/%s.sort.bam O=$BAM/%s.sort.dedup.bam M=$BAM/%s.marked_dup_metrics.txt REMOVE_DUPLICATES=true CREATE_INDEX=true VALIDATION_STRINGENCY=LENIENT.

GATK: HaplotypeCaller (-ERC GVCF), GenotypeGVCFs (-stand_call_conf 10 -variant_index_type LINEAR -variant_index_parameter 128000) and SelectVariants (--excludeNonVariants -restrictAllelesTo BIALLELIC -selectType SNP or INDEL).

PLINK: ROH (--homozyg --allow-no-sex --homozyg-window-snp 50 --homozyg-window-het 1 --homozyg-window-missing 5 --homozyg-density 50 --homozyg-kb 1000 --homozyg-snp 100 --chr-set 29).

Beagle: imputed missing alleles (java -jar beagle.27Jul16.86a.jar chrom=$chr gtgl=$chr.vcf.gz out=$chr.imp gprobs=true) and inferred haplotype (java -Xmx50g -Xss128m -jar beagle.27Jul16.86a.jar gt=$chr.imp.vcf.gz out=$chr.imp.phase gprobs=true).
